# Context-acceptability theories: example of family planning interventions in five African countries

**DOI:** 10.1186/s13012-020-01074-z

**Published:** 2021-01-12

**Authors:** Jayne Webster, Shari Krishnaratne, Jenna Hoyt, Shiferaw Dechasa Demissie, Nathaly Spilotros, Justine Landegger, Misozi Kambanje, Shannon Pryor, Easterlina Moseti, Seth Marcus, Marius Gnintoungbe, Dora Curry, Jessie K. Hamon

**Affiliations:** 1 Disease Control Department, London School of Hygiene and Tropical Medicine, London, UK; 2 International Rescue Committee Ethiopia Programme, Addis Ababa, Ethiopia; 3 International Rescue Committee US, New York, USA; 4 Save the Children Malawi Country Office, Blantyre, Malawi; 5 Save the Children US, Washington, DC USA; 6 World Vision International, Nairobi, Kenya; 7 World Vision US, Monrovia, CA USA; 8 CARE Benin, Cotonou, Benin; 9 CARE USA, Atlanta, USA

**Keywords:** Context-acceptability theory, Realist evaluation, Family planning

## Abstract

**Background:**

Family planning (FP) can lengthen birth intervals and potentially reduce the risk of foetal death, low birthweight, prematurity, and being small for gestational age. Effective FP is most easily achieved through access to and acceptability of modern contraceptive methods (MCMs). This study aimed to identify mechanisms of acceptability and the contexts in which they are triggered and to generate theories to improve the selection and implementation of effective interventions by studying an intervention integrating FP with childhood immunisation services.

**Methods:**

Qualitative interpretative synthesis of findings from realist evaluations of FP interventions in five African countries was guided by an analytical framework. Empirical mechanisms of acceptability were identified from semi-structured interviews and focus group discussions with key stakeholders (*N* = 253). The context in which these mechanisms were triggered was also defined. Empirical mechanisms of acceptability were matched to constructs of a theoretical framework of acceptability. Context-acceptability theories (CATs) were developed, which summarised constructs of acceptability triggered for specific actors in specified contexts. Examples of interventions that may be used to trigger acceptability for these actors were described.

**Results:**

Seven CATs were developed for contexts with strong beliefs in religious values and with powerful religious leaders, a traditional desire for large families, stigmatisation of MCM use, male partners who are non-accepting of FP, and rumours or experiences of MCM side effects. Acceptability mechanisms included alignment with values and beliefs without requiring compromise, actors’ certainty about their ability to avoid harm and make the intervention work, and understanding the intervention and how it works. Additionally, acceptability by one group of actors was found to alter the context, triggering acceptability mechanisms amongst others.

**Conclusions:**

This study demonstrated the value of embedding realist approaches within implementation research. CATs are transferable theories that answer the question: given the context, what construct of acceptability does an intervention need to trigger, or more simply, what intervention do we need to apply here to achieve our outcomes? CATs facilitate transfer of interventions across geographies within defined contexts.

**Supplementary Information:**

The online version contains supplementary material available at 10.1186/s13012-020-01074-z.

Contributions to the literature

- Acceptability is key to the successful implementation of interventions, and interventions work in some contexts but not in others. However, the relationship between context and acceptability is poorly explored within the literature.
- We demonstrate how different contexts trigger mechanisms of acceptability, and we explain the influence of actors and interventions on this relationship. Our findings highlight the non-static nature of both contexts and mechanisms of acceptability in the implementation of interventions.
- We generate novel context-acceptability theories and recommend their use for identifying intervention implementation needs in specified contexts.

## Background

A child born to a woman less than 18 months after the birth of her previous child has an increased risk of foetal death, low birthweight, prematurity, and being small for gestational age [1, 2]. Ensuring that no child is born within a birth interval of less than 2 years could avert approximately 1 million of the 11 million deaths per year of children under 5 years of age [3]. Although modern contraceptive methods (MCMs) (condoms, oral contraceptive pills, injectables, implants, and the intrauterine device, excluding traditional/natural methods) could help lengthen birth intervals, 218 million women in low- and middle-income countries who are of reproductive age and want to avoid pregnancy are not using MCMs [4]. The use of MCMs, as with the use of public health interventions more generally, requires access to and acceptability of the intervention by healthcare workers (HCWs) and recipients.

Family planning (FP) studies have explored the acceptability of specific MCMs such as intrauterine devices (IUDs) [5], female condom [6], male condom shape [7], self-injections [8], and contraceptive vaginal rings [9, 10] as well as behaviour change methods to increase FP uptake including mobile FP decision aids [11] and mobile phone behavioural interventions [12]. FP studies have also investigated the delivery of interventions by different cadres of facility- and community-based health workers such as community health workers (CHWs) delivering the standard days method [13] and community extension workers delivering subdermal implants [14]. However, acceptability is rarely defined in these studies despite commonly being implied through the measurement of quantitative outcomes or through the interrogation of qualitative themes.

Acceptability is an ambiguous term with its defined meaning varying from ‘welcome, or pleasing’ to ‘barely satisfactory or adequate’ [15, 16]. In terms of FP services and MCMs, the meaning of acceptability could vary between, for example, being very pleased with the ability to space births using an IUD and needing to hide the use of MCMs from a husband or other community members. In 2017, Sekhon et al. advanced the field of research on acceptability by defining acceptability as ‘a multi-faceted construct that reflects the extent to which people delivering or receiving a healthcare intervention consider it to be appropriate, based on anticipated or experienced cognitive and emotional responses to the intervention’ [17]. This definition suggests that the acceptability of an intervention is influenced by the attributes of the intervention and how it is delivered, but also by the reaction to the intervention by providers and users. An individual’s reaction to an intervention may be due to their own past or anticipated experiences and to their own emotions. Likewise, their reaction can also be due to their expectations of how their peers or members of their community will react.

The influence of an individual’s emotional response on his or her reaction to a given intervention resonates with the concept of mechanisms within realist approaches to research and evaluation [18]. In the field of realist research, mechanisms are the decisions, or reasoning, that people make in response to available resources. Realist evaluation aims to identify the mechanisms that drive the outcomes of an intervention and is based on the assumption that ‘the intervention does not work directly to cause an outcome, but via a mechanism that is triggered in some contexts and not in others’ [19].

Context interacts with mechanisms in a way that either enables or constrains them and that ultimately determines the direction (positive or negative) of an outcome. Context may be defined as the ‘prevailing beliefs, social and cultural norms, regulations and economic factors’ [20] or, in implementation science, as ‘the characteristics of the conditions in which the interventions are introduced’ [18] and is therefore multi-layered, multi-dimensional, and fluid. Both context and mechanisms can modify or be used to modify the logic of an intervention, that is, how an intervention works or was designed to work. They can also help to identify when an intervention is likely to work and be most effective.

This paper aims to identify mechanisms of acceptability and the contexts in which they are triggered and to generate theories to aid in improving the selection and implementation of effective interventions through the study of an intervention integrating FP with childhood immunisation services.

## Methods

### Study sites

The study took place in sites of varying size ranging from 14 to 114 health facilities (hospitals, health centres, and health posts) and 24 routine outreach clinics in five countries. Each study site encompassed a project on integrated delivery of FP and childhood immunisation services led by a non-governmental organisation (NGO). The study sites were Adjohoun-Bonou-Dangbo district, Ouémé department, Benin; Assosa and Bambasi woredas, Benishangul-Gumuz region, Ethiopia; Garba Tulla, Isiolo county, and Pokot West and Pokot South, West Pokot county, Kenya; Mwanza, Blantyre, and Thyolo districts, Malawi; and Karamoja sub-region, Uganda.

Unmet need for FP^1^ amongst married women varied across countries according to the latest Demographic and Health Surveys (DHS) at 33.7% in Ouémé department, Benin [21]; 21.1% in Benishangul-Gumuz region, Ethiopia [22]; 12.4% in Eastern region and 20.8% in Rift Valley region, Kenya [22]; 20.3% in Southern region, Malawi [23]; and 19.7% in Karamoja region, Uganda [24]. These data, however, mask intra-regional and intra-district variations in unmet need for FP.

### Intervention

All project interventions had the shared aim of increasing the use of MCMs amongst postpartum women by integrating the delivery of FP services with childhood immunisations through the Expanded Programme on Immunisation (EPI). The components of the intervention varied by country based on the structure and function of the health system and on the socio-cultural context. Generally, the interventions involved a training component for health facility staff and community health workers; information, education, and communication (IEC) at the community level; and referrals from immunisation to FP services. Delivery points for FP services were health facilities (Benin, Kenya, Uganda), health posts and home visits (Ethiopia), and outreach clinics (Malawi). Where FP services were delivered at health facilities, both FP counselling and administration of MCMs were conducted by midwives and/or nurses. FP counselling and MCM administration at health posts, outreach clinics, and via home visits were through different cadres of community health workers, health extension workers in Ethiopia, and health surveillance assistants in Malawi. In addition, peer influencers were active at the community level to mobilise women to attend FP and immunisation services in Benin, Ethiopia, Kenya, and Uganda.

The interventions were designed and implemented by NGOs: Care in Benin, the International Rescue Committee in Ethiopia and Uganda, World Vision in Kenya, and Save the Children in Malawi.

### Study design

This study was a qualitative interpretive evidence synthesis [25] of stakeholder interview data from a realist evaluation of integrated FP and childhood immunisation services. Whilst there are a plethora of research studies describing the prevalence of, and socio-demographic associations with, the use of MCMs in sub-Saharan Africa, the mechanisms driving the acceptability and adoption of these methods are less well understood. Resonance between concepts of acceptability and realist mechanisms suggests that a realist approach to evaluation could be useful in understanding the acceptability of FP (the spacing and limiting of births) and of the MCMs supported by the interventions (i.e. contraceptive methods excluding traditional and natural methods). The realist evaluation methods were described by Krishnaratne S, Hamon JK, Hoyt J, Chantler T, Landegger J, Spilotros N, et al: What mechanisms drive uptake of family planning when integrated with childhood immunisation in Ethiopia? A realist evaluation, unpublished. For each individual country, the realist evaluation included the development of an initial programme theory, which presented perceptions of what was driving the success of the intervention, as well as the potential barriers. These theories were developed in a workshop with programme designers and implementers from each country, approximately 15 months after the start of implementation.

### Data collection and management

Semi-structured interviews (SSIs) and focus group discussions (FGDs) were conducted in each country between October 2017 and March 2018 with stakeholders who were either directly or peripherally involved in the projects. Stakeholders with a range of perspectives and opinions on the projects were purposively selected [26] and included implementing partners, government officials, HCWs, community leaders, and women. HCWs were from project sites that were performing well and sites that were performing less well according to monitoring data. SSI and FGD guides were developed to include broad themes on workload, socio-cultural context, healthcare access, reasons for use or non-use of MCMs, barriers to MCMs use, access to FP services, and community-level acceptance of MCM use, together with questions focussed on components from an initial programme theory.

Interviews were conducted in each country by SK together with a trained local interviewer who was conversant in local languages. In Benin, interviews were conducted in French and Ouémé; in Ethiopia, in Amharic and English; in Kenya, in Borana, Pokot, and English; in Malawi, in Chichewa and English; and in Uganda, in Karamojong and English. All interviews were audio recorded, transcribed *verbatim*, and then translated into English (if needed) by experienced transcribers and translators.

### Analytical framework

**Fig. 1:**
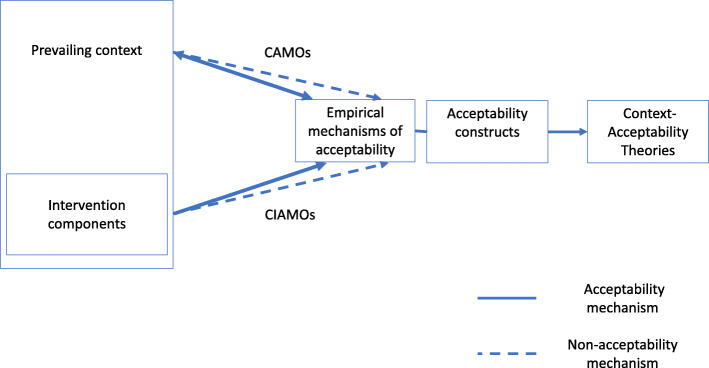
Analytical framework

An analytical framework was developed to guide the analysis and was based on realist and acceptability concepts. First, the framework conceptualised relationships between context and intervention components in triggering empirical mechanisms of acceptability. Our analytical framework assumed that the prevailing context, that is the conditions in which the interventions were introduced [18], could directly influence mechanisms of FP acceptability amongst women and the community (including acceptability and non-acceptability); that the context provided the conditions in which interventions triggered mechanisms; and that the intervention could influence and change the prevailing context (Fig. 1). The framework presents context-actor-mechanism-outcome (CAMO) configurations and context-intervention-actor-mechanism-outcome (CIAMO) configurations [27] as the triggers of the empirical mechanisms of acceptability directly by the prevailing context and by the intervention within the prevailing context, respectively.

Second, the analytical framework matches empirically derived mechanisms of acceptability to the constructs of Sekhon et al.’s Theoretical Framework of Acceptability (TFA) [17], ultimately producing context-acceptability theories (CATs). The TFA consists of seven constructs of acceptability relating to participants’ perceptions and feelings about the intervention: affective attitude (general feelings about the intervention), burden (perceptions of effort needed to take part), ethicality (fit with their value system), intervention coherence (understanding of the intervention and how it works), opportunity costs (what must be given up in order to participate), perceived effectiveness (perceptions of the intervention’s ability to achieve its aim), and self-efficacy (feeling that they are able to do what they need to do to take part). The TFA was chosen as the theoretical basis for the development of CATs as it provided clearly defined building blocks for theorising through its seven constructs [28], potential to explore mechanisms triggering constructs of acceptability, and links between triggers of acceptability constructs and context. The CATs developed through this analytical framework are middle-range theories. Middle-range theories enable the consolidation of hypotheses and empirical realities and allow these to be grouped into theories that can be further empirically tested [29].

### Data analysis

Translated transcripts were imported into NVivo 11.2 for coding and analysis. Data was anonymised and labelled by type of stakeholder. A coding framework was developed which included context (economic/political, community/social, and organisational/team), actors (community members and HCWs), interventions (integration of immunisation and FP, community mobilisation, HCW training, and supply chain systems), mechanisms (HCW decision-making, community decision-making, women’s decision-making), and outcomes (expected and unexpected/unrelated or additional). This deductive coding was supplemented by the inductive addition of sub-codes, which expanded the depth of information within each code. Coding was undertaken for each country individually.

An initial thematic narrative analysis [30] was conducted for the realist evaluation for each of the five countries using the individual country coding and further analysis to construct context-mechanism-outcome configurations (CMOs) [18]. The CMOs for each country were used to construct a revised programme theory to represent the context- or intervention-related triggers of identified mechanisms, which were perceived by stakeholders to drive intermediate outputs and ultimately the outcome of FP uptake in each country. CMOs and revised programme theories were then discussed and validated by co-authors (JKH, JH, and SK).

For the context-acceptability synthesis, the realist evaluation CMOs were re-examined to identify those that included mechanisms of acceptability, and expanded to either CAMOs or CIAMOs. CAMO configurations were used in this study to analyse the influence of the prevailing context on mechanisms of acceptability and CIAMO configurations to analyse the influence of the intervention on mechanisms of acceptability. Identified empirical mechanisms of acceptability were then reviewed against and matched to TFA constructs of acceptability. Adaptations to the TFA constructs were made as required.

Finally, CATs that summarised constructs of acceptability triggered for specific actors in specific contexts were developed and examples of interventions that could be used to trigger acceptability for these actors were described. The Standards for Reporting Qualitative Research [31] and reporting which incorporate the role of theory were used to ensure rigorous reporting of the study (see additional information).

### Ethics

Ethics approval was obtained from the Comité d’Éthique de l’Institut des Sciences Biomédicales Appliquées, Benin; Benishangul-Gumuz Regional Ethical Review Board, Ethiopia; African Medical and Research Foundation, Kenya; National Committee on Research in the Social Sciences and Technology, Malawi; Mildmay Uganda Research Ethics Committee, Uganda; and the ethics committee of the London School of Hygiene & Tropical Medicine.

## Results

A total of 94 SSIs and 21 FGDs were conducted (*N* = 253 participants) with women (MCM users and non-users), HCWs (delivering FP and childhood immunisation services), health administrators and implementing partners, community members (including religious leaders and community health volunteers), and male community members (Table 1).

**Table 1:** Interviews and focus group discussions by participant groups

	Women (FP users and non-users)	HCWs^a^ (midwives, nurses)	Health administrators/implementing partners	Community members (religious leaders, health volunteers)	Male community members
**Benin**	3 FGDs (*N* = 20)	6 SSIs	4 SSIs	None	2 FGDs (*N* = 10)
**Ethiopia**	1 SSI	7 SSIs	6 SSIs	9 SSIs	None
**Kenya**	4 FGDs (*N* = 43)	3 SSIs	5 SSIs	4 SSIs and 5 FGDs	2 FGDs
**Malawi**	7 SSIs	3 SSIs and 4 FGDs (*N* = 25)	10 SSIs and 1 FGD (*N* = 3)	2 SSIs	None
**Uganda**	5 SSIs	10 SSIs	5 SSIs	7 SSIs	None
	76	54	33	65	25

^a^This includes HSAs for Malawi because they delivered the intervention in routine outreach clinics

### The prevailing context and mechanisms of acceptability of FP/MCMs: CAMOs

Five major prevailing contexts were found to directly influence the mechanisms of acceptability of FP by the community, men, and women (Table 2). These were (1) FP was believed to be incongruous with Islamic (Ethiopia and Kenya) and Christian (Kenya) religious principles, teachings, and values; (2) traditional desire for large families and the assumption that FP aims to reduce the family size (Kenya, Uganda); (3) stigmatisation of MCM use (Benin); (4) some male partners who are non-accepting of FP or MCM use (all countries to differing extents); and (5) experience or rumours of side effects of MCMs (all countries). Each of these contexts directly led to mechanisms of non-acceptability of FP or MCMs amongst specific actors.

**Table 2:** Major contextual factors and context-actor-mechanism-outcome configurations

Context	CAMO	Acceptability construct
Strong belief in religious values	**CAMO 1**: Strong belief in religious values (C) such that the community (A) consider FP to be out of line with religious principles or forbidden (M) and do not accept FP (O)	Ethicality
Respondent (R): ‘They say that if you die and the implant is still within you then it is against the Muslim religion, as you are not supposed to be buried with any foreign object inside you. They also say preventing a baby is forbidden’. Ethiopia Nurse_23 R: ‘Am a Catholic by faith so before when….I was growing as a child I was told and heard about family planning as a bad thing’. Kenya Religious leader_10		
Traditional desire for large family and belief that FP aims to limit family size	**CAMO 2:** Traditional desire for large family and belief that FP aims to limit family size (C) such that the community (A) rejects FP as being against their traditional values (M) and FP is not accepted by the community (O)	Ethicality
R: ‘Within the community, family planning is stigmatised in the way it was introduced before. They say family planning is to have few number of children which the people don’t want that. You are trying to control the number because our people say people is wealth. You have big wealth when you have more people…’ Kenya Health administrator19 R: ‘And then also polygamy, when a man has many women, they compete to have children, you know to own a man. They think that you win a man by overproducing’. Uganda Midwife_7		
MCM use is stigmatised	**CAMO 3:** Stigmatisation of MCM use and belief that it is linked to prostitution (C) lead men (A) to fear their wives will be seen as prostitutes (M) and to their non-acceptance of MCM use (O)	Unintended consequences
R: ‘It is bad in the context of certain women getting the family planning method, making use of the protection that the planning gives them, to take up prostitution. That is where we as men start being reluctant’. Benin Men’s FGD_15 R: ‘The women do not want the men to know that they have done the family planning because the men think that a woman who has agreed to family planning is more likely to become a prostitute. Therefore, for men, a woman who is using family planning can prostitute herself and cannot be controlled’. Benin Midwife_5		
Male partners are non-accepting of FP or MCM use	**CAMO 4:** Male partners are non-accepting of FP or MCM use (C) and women (A) feel that they cannot use MCMs or that they have to hide their MCMs (M) so women do not openly accept MCMs (O)	Self-efficacy
R: ‘Most people say…. men do not allow their wives to go for family planning. Some even hide their health passports so that their husbands should not know that they are on family planning. Some wait for their husbands to go to work so that they should go to the clinic and access family planning’. Malawi Woman FP user_14 R: ‘Most of them use the injectable one because of the husbands. For example if they use implants that means husband will discover – then problem. If they take pill then the same, husbands will discover – problems...’. Malawi Nurse/FP trainer_22		
Experience or rumours of side effects of MCMs	**CAMO 5:** Women have experience with or hear rumours of side effects of MCMs (C) such that women (A) are afraid of side effects (M) and do not accept MCMs (O)	Unintended consequences
R: ‘some of the challenges we get in the village are that when one person using family planning develop side effects and maybe that person was sick and so many others will see family planning as bad’. Kenya CHV FGD_15 R: ‘The women in the village refuse because they say family planning blocks stomachs and it makes you barren hence you can’t produce again. That family planning removes feelings for sleeping with your husband. That a woman who practices family planning will become a prostitute’. Uganda Women’s empowerment group member_18		

### Context and project intervention components driving mechanisms of acceptability and non-acceptability: CIAMOs

Nine CIAMOs representing the empirical mechanisms of acceptability triggered by intervention components in specific actors within each context were identified, as was one CIAMO representing an empirical mechanism of non-acceptability (Table 3).

**Table 3:** Contexts, interventions, actors, mechanisms, and constructs of acceptability

Context	Intervention components	Actor, empirical mechanism of acceptability, and outcome	Acceptability construct
**CIAMO 1:** Strong belief in religious values	Analysis of religious text together with religious leaders (I)	Religious leaders (A) recognise and agree that FP fits with their religious values (M) and accept FP (O)	Dominant: Ethicality Additional: Opportunity costs
**CIAMO 2:** Traditional desire for large family	Focus FP messages on birth spacing rather than limitation (I)	Community (A) recognise and agree that spacing births does not have to compromise their family size aspirations (M) and accept FP (O)	Dominant: Ethicality Additional: Opportunity costs, intervention coherence
**CIAMO 3 and 4:** MCM use stigmatised	Referral cards from childhood immunisations to FP services (I)	Women (A) fear the visibility of accessing FP services due to referral cards (M) and do not accept MCMs (O)	Dominant: Unintended consequences
	Provide access to MCMs in private (I)	Women (A) feel confident to accept MCMs when these MCMs are not visible to their peers (M) and accept MCMs (O)	Dominant: Self-efficacy and unintended consequences Additional: Opportunity costs
**CIAMO 5, 6, and 7:** Male partners non-accepting of FP or MCM use	Male role models promote financial, educational, and health benefits of FP/birth spacing (I)	Male partners (A) relate to and trust male role models and their messages (M) and therefore believe the messages (M) and accept birth spacing with MCMs (O)	Dominant: Intervention coherence Additional: Perceived effectiveness
	Religious leaders promote the alignment of FP with religious values (I)	Male partners (A) respect and trust religious leaders (M) and therefore believe in alignment of FP with religious values (M) and accept FP (O)	Dominant: Ethicality Additional: Opportunity costs
	Integrated FP and childhood immunisation services (I)	Women (A) feel that they are able to hide their use of FP services and have autonomous decision-making (M) and accept MCMs (O)	Dominant: Self-efficacy Additional: Opportunity costs
**CIAMO 8 and 9:** Experience of or rumours of side effects of MCMs	Train HCWs and expert clients in the community to discuss side effects (I)	Women (A) feel they can talk to HCWs about side effects and seek advice on their management (M) and accept MCMs (O)	Dominant: Self-efficacy Additional: Unintended consequences
	Message layering (I)	Women (A) are convinced by messages having received them multiple times via multiple HCWs and CHWs (M) and accept MCMs (O)	Dominant: Self-efficacy Additional: Intervention coherence

#### CIAMO 1

Interventions to counteract the religion-related non-acceptability of FP were perceived as successful in both Ethiopia and Kenya. In Ethiopia, Islamic religious leaders were well respected and powerful within the project sites, and FP was considered *Haram*, or forbidden, by religious law. In both countries, the study of religious texts was conducted using the Koran with Islamic leaders and the Bible with Christian leaders. This involved highlighting passages that illustrated alignment with the concept of FP (spacing or limiting births). This exercise convinced the religious leaders involved that FP aligned with their religious principles. Further IEC activities involving these leaders and others within their congregations helped to spread this message.

#### CIAMO 2

Resistance to the idea of limiting family size, especially amongst men, based on both religious and traditional values, was particularly strong in project sites in Kenya and Uganda. The intervention in these countries promoted messages about MCM use that focused on birth spacing rather than limiting family size. Additionally, advocates, including male champions and male role models, were used to convince men of the financial, educational, and health-related advantages of birth spacing.

#### CIAMO 3

In Benin, MCMs were strongly stigmatised, with men in particular linking MCM use to women engaging in prostitution. They also believed MCM use would lead to the loss of power over their wives. This belief meant that women did not want to be seen accessing FP services by their peers for fear it would get back to their husbands. The stigma, and related fear, meant that the referral cards given to women from childhood immunisation services to access FP services were perceived negatively by women as it resulted in increased visibility. To address this, referral cards were eventually withdrawn from the intervention.

#### CIAMO 4

Due to the stigma of MCM use, women and HCWs in Benin adapted their behaviour to enable discreet engagement with FP services. Some women who initially refused FP services returned later at night or after clinic hours to ask for a consultation and uptake of MCMs, which was facilitated by the HCWs.

#### CIAMO 5

In Kenya, where some male partners were non-accepting of FP, the intervention included the promotion of the financial, educational, and health benefits of FP by male role models. These male role models included men with as many as twelve children, who were able to speak to the challenges of providing for a large family, often exacerbated by insufficient spacing between births. Male partners both related to and trusted the male role models and therefore believed their messages.

#### CIAMO 6

Following the acceptance of FP by religious leaders, some leaders openly promoted FP and MCM use not just in their religious institutions but also openly at meetings in communities. This acceptance by religious leaders became the new prevailing context in which the project operated. Again, following the religious leaders’ and male partners’ support for FP, it was much easier for women to begin using MCMs as they were confident that they would be supported.

#### CIAMO 7

In Malawi, the project integrated the delivery of FP with childhood immunisation services in outreach clinics, which helped women hide their use of FP services from male partners. This meant that in the context where some male partners were non-accepting of FP, women were able to make autonomous decisions about MCM use without the knowledge of their male partners.

#### CIAMO 8

Experiences or rumours of MCM side effects were mentioned by respondents in all countries. Training HCWs not only to build their knowledge about side effects, but also to equip them with the skills needed to discuss side effects with women were perceived to be important. In Uganda, the intervention included a mentorship programme to help HCWs manage side effects, as well as the training of expert clients in the community who were able to dispel myths about MCM use and with whom women felt able to discuss MCMs.

#### CIAMO 9

Message layering about FP, that is the delivery of messages on more than one occasion and often through different HCWs or other individuals involved in IEC activities, was perceived as an effective way of convincing women of the benefits of FP in Benin. At the community level in Uganda, message layering was perceived as a way of priming women for them to be ready to accept MCMs when attending a health facility.

### Mechanisms and constructs of acceptability

Empirical mechanisms of acceptability were aligned to one or more constructs of the TFA and varied by context and actor (Table 3). Mechanisms that were assessed to be strongly aligned to a specific construct were termed dominant, and mechanisms that were more tenuously aligned to other constructs were termed additional. Ethicality was a dominant mechanism of acceptability of FP in contexts where there was a strong belief in religious values and a traditional desire for large families. Ethicality was accompanied by opportunity costs such that leaders understood that they would not need to compromise on their religious values to accept, promote, and encourage the use of MCMs. In contexts where a traditional desire for large families was prevalent, mechanisms of acceptability were aligned with these same constructs of ethicality and opportunity costs but in the community more generally, including amongst women. That is, using MCMs to achieve healthy birth spacing was no longer equated with compromising on the desired family size. Acceptance of birth spacing also aligned with the construct of intervention coherence and was extended beyond the TFA definition to include ‘believing information on how it worked’.

The TFA was adapted to include an additional construct: ‘unintended consequences’ which was defined as ‘the extent to which the intervention leads to consequences beyond those for which it was designed’. The elevated risk of stigmatising women was perceived as an unintended consequence of the use of referral cards from childhood immunisation to FP services in Benin, due to women’s fears of being seen accessing FP services by their peers. Providing access to MCMs in private after the clinic closed was a reactive adaptation of the intervention led by HCWs to the stigmatisation of MCM use. This mechanism of women feeling confident to accept MCMs when they were not seen by others aligned with the construct of self-efficacy, as women were confident they could do what was needed to take part in the intervention without the risk of unintended consequences.

Intervention coherence was a dominant TFA construct aligned to the empirical mechanisms driving acceptability of FP and MCM use amongst male partners. At intervention sites in Kenya, male partners believed the information they received about the advantages of birth spacing, in terms of financial, educational, and health benefits for their families, because this information was provided by trusted and relatable male role models. Male partners also acknowledged the value of using MCMs to space births, which was perceived effectiveness.

### Context-acceptability theories

Seven CATs were developed by linking the empirically derived mechanisms of acceptability to TFA constructs. Three of the theories involved ethicality mechanisms of acceptability, three involved self-efficacy (with or without unintended consequences), two included opportunity costs, and one involved intervention coherence and unintended consequences. In Table 4, each CAT includes an identified context and the mechanism that will lead to the acceptability of FP (and MCM use) within this context. Thus, a CAT specifies what exactly will trigger an actor to accept FP and therefore highlights the response in the actor that an intervention needs to bring about. Conversely, a CAT also indirectly suggests what interventions are not likely to be successful. For example, in contexts where there is a strong belief in religious values and where there are powerful religious leaders, an intervention that only teaches religious leaders about how FP works and about its benefits to women and families is unlikely to be effective as it will not trigger acceptability mechanisms aligned with ethicality, i.e. religious values (theory 1). Similarly, telling women that they may experience side effects when using MCMs without teaching them how to manage these should they occur is unlikely to be effective as acceptability mechanisms aligned with self-efficacy will not be triggered (theory 7).

**Table 4:** Context-acceptability theories

Theory 1	**In contexts of strong belief in religious values and powerful religious leaders** **Religious leaders’ recognition that FP aligns with these values and they do not need to compromise on their values will lead to their acceptability of FP.** An example of an intervention is the examination of religious text to identify passages aligning with FP. *Mechanisms of acceptability: ethicality and opportunity costs*
Theory 2	**In contexts of strong belief in religious values and of powerful religious leaders** **Men’s trust in religious leaders’ belief that there is alignment between FP and religious values will lead to acceptability of FP by men**. An example intervention is religious leaders promoting the alignment of religious text with FP. *Mechanism of acceptability: ethicality*
Theory 3	**In contexts of traditional desire for a large family** **The community’s recognition that FP does not require a compromise of their family size aspirations will lead to their acceptability of FP.** An example intervention is the focussing of messages and trainings on birth spacing. *Mechanisms of acceptability: ethicality and opportunity costs*
Theory 4	**In contexts where MCMs are stigmatised** **Providing the potential for women to feel that they can access MCMs discretely will lead to their acceptability.** Example interventions are providing access to FP services outside of clinic hours and avoiding presenting women with written notification of their attendance, such as referral cards. *Mechanism of acceptability: self-efficacy and opportunity costs*
Theory 5	**In contexts where male partners are non-accepting of FP or MCM use** **Providing the potential for women to feel that they can access MCMs discretely will lead to their acceptability.** An example intervention is the co-delivery of immunisation and FP services. *Mechanism of acceptability: self-efficacy*
Theory 6	**In contexts where male partners are non-accepting of FP or MCM use** **Men’s recognition of the financial, educational, and health benefits of using MCMs for birth spacing will lead to the acceptability of MCM use when they trust those delivering the messages.** An example intervention is using male role models to deliver messages on the financial, educational, and health benefits of MCMs. *Mechanism of acceptability: intervention coherence and self-efficacy*
Theory 7	**In contexts where there are rumours or experience of MCM side effects** **Women need to feel that they can manage potential side effects. Ensuring that there are trained HCWs and community health workers that women can talk to about potential side effects will lead to women’s acceptability of MCMs.** An example intervention is the training of expert clients and satisfied family planning users from the community. *Mechanism of acceptability: self-efficacy*

### Context-acceptability cascades

**Fig. 2:**
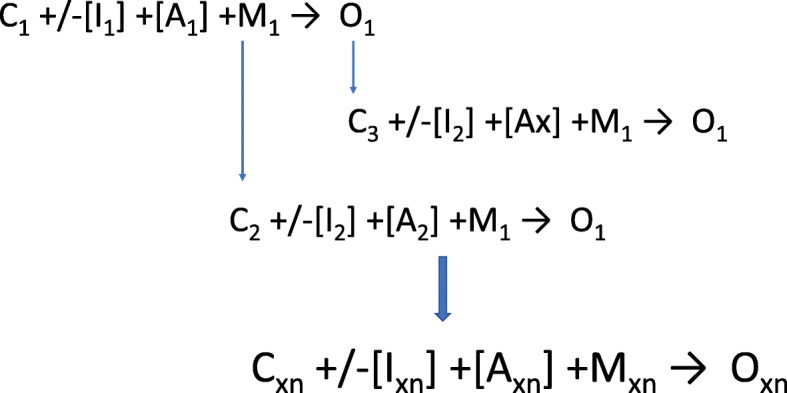
Context-acceptability cascade. Using the example of religious leaders in Kenya: in an initial prevailing context of strong religious beliefs and powerful religious leaders (C_1_), examination of religious text for alignment with FP (I_1_) with religious leaders (A_1_) triggers recognition that FP aligns with religious values (M_1_), leading to acceptance of FP (O_1_). The recognition that FP aligns with religious values is a new context (C_2_) which may itself or with advocacy activities of religious leaders (I_2_) trigger recognition of alignment of religious text and FP (M_1_) in men (A_2_) leading to acceptance of FP (O_1_). Similarly, the acceptance of FP by religious leaders leads to a new context (C_3_) with initiation of a similar process as that initiated by (C_2_). Generally, project/programme conditions represent a number of differing prevailing contexts (Cxn), which in the presence or absence of an intervention/s (Ixn) trigger mechanisms (Mxn) in different actors (Ax) and lead to a number of potential outcomes (Oxn). Using empirical data to elucidate and describe these context-acceptability cascades enables deeper understanding of the fluidity of the interplay between context, interventions, mechanisms, and outcomes

The acceptance of FP alignment with religious values and the promotion of this alignment in the community created a new prevailing context for men in Kenya as they came to perceive FP as fitting with their values (ethicality). Subsequently, acceptance of FP amongst male partners became the new prevailing context in which women felt they were supported and therefore able to accept and use MCMs (self-efficacy). This can be seen as a context-acceptability cascade where mechanisms and outcomes of acceptability in one group of actors, or an intervention, change the prevailing context for another group. This new prevailing context then in turn triggers the second group’s mechanisms of acceptability (Fig. 2). The CAT for each population group within this cascade differs.

## Discussion

This study identified (1) prevailing contexts that directly triggered mechanisms of acceptability of FP/MCMs, (2) interventions that triggered mechanisms of acceptability within categorised contexts, and (3) the actors for whom these contexts and interventions triggered mechanisms of acceptability. This study also linked empirically derived mechanisms of acceptability with TFA constructs to develop CATs, which are transferrable middle-range theories. The identification of these empirical mechanisms of acceptability and their CATs needs to be an ongoing process during intervention implementation. That is, theory development should be a process throughout the life of a project rather than used purely at intervention design and/or final evaluation stages [32].

### The prevailing context and mechanisms of acceptability

The mechanisms of acceptability that were identified from the empirical data and that were directly triggered by the prevailing context centred on contexts with a negative or potentially negative influence on the acceptability of FP/MCMs. For each of the five categories of prevailing contexts, intervention components were identified that triggered positive mechanisms of FP acceptability despite the negative contextual influences. Some intervention components targeted specific actors and others were aimed at the wider community. Interestingly, there were instances where it was not only the intervention itself that triggered a mechanism of acceptability, but also the actor who delivered it. For example, where male role models were used to promote the financial, educational, and health benefits of using MCMs for birth spacing, mechanisms of trust in, and relating to, the male role models who delivered the messages contributed to the mechanism of belief in the messages. Findings from this study support the idea that context is not only multi-layered, but is also non-static. Context may change during the implementation of an intervention due to external factors or due to components of the intervention itself. This change in context will lead to new mechanisms of acceptability.

### Matching empirical mechanism of acceptability and the TFA

Knowledge of empirical mechanisms is of practical value in both the design and ongoing implementation of interventions. For instance, during the design of an intervention, identifying that in some strong religious contexts FP and the use of MCMs are believed to be misaligned with religious values highlights what the trigger for acceptability will be. The identification of these triggers, and of their nature, indicates what interventions should focus on and which population group they should target to be effective.

Matching empirical mechanisms of acceptability with TFA constructs allows for abstraction and transferability. The middle-range CATs identify which mechanism of acceptability must be triggered in a given context. For example, in contexts of strong religious values, ethicality is the mechanism of acceptability that needs to be triggered. Therefore, the intervention that is chosen must be able to convince actors that FP fits with their values. Showing alignment of FP using religious text was an example of this. Similarly, there may be other interventions that can convince key actors that MCM use does not compromise their values. However, it is unlikely that an intervention that only focusses on the effectiveness of FP and/or how it works would achieve an increase in acceptability of FP in this context. This approach to identifying dominant constructs of acceptability may be useful in increasing the acceptability of other public health interventions beyond FP.

### Novel FP strategies requiring further evidence

Our CATs identified five major contexts impacting the acceptability of FP with nine intervention components triggering mechanisms of acceptability in these contexts. Whilst these include relatively recognised FP strategies (e.g. birth spacing messaging), further evidence on intervention outcomes and processes would be valuable for other, more novel, strategies. The relationship between religious values and FP varies across religions and in denominations and sects within these religions [33, 34]. Christian texts and Islamic texts without direct statements can lead to varied interpretation in both support and opposition to FP [35] and may be driven by the views of individual local religious leaders [36]. This adds support for the potential of well-considered interpretation of religious texts, and of exploration into the most effective processes for disseminating these interpretations.

Privacy and covert use of MCMs fit naturally within the women’s empowerment agenda and associated frameworks [37, 38] and research; however, the majority of the studies on women’s sexual and reproductive empowerment have been conducted outside of sub-Saharan Africa [39, 40]. Recent studies in a number of countries of East and West Africa have investigated women’s reasons for covert use, challenges to covert use, and consequences of discovery [41–44]. Realist approaches to interrogating decision-making offer opportunities to deepen the understanding of women’s covert use of MCMs within currently developed women’s empowerment frameworks of the existence of choice, exercise of choice, and achievement of choice [40]. Management of side effects of MCMs is a challenge to continued covert use [41] and is an important component of FP counselling. An understanding of the influence of advice given on side effect management and of the ability to manage side effects on the continuation of MCM use amongst women who are covertly using MCMs would be important in supporting women who are unable to openly use MCMs.

### Applicability of the TFA in developing CATs

**Fig. 3:**
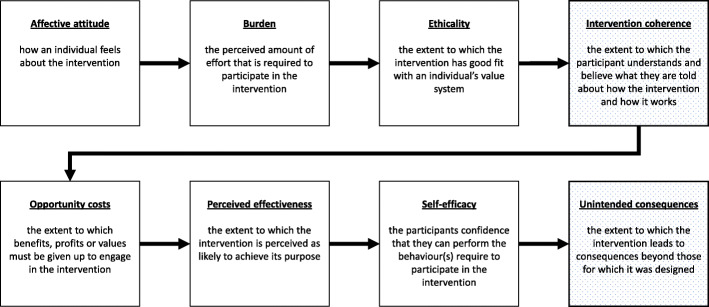
Adapted TFA constructs. Source: Sekhon et al. [17]*;* adaptation = shaded constructs

Multiple theories have been used in FP studies; however, to our knowledge, the TFA has not been used. For example, the Theory of Planned Behaviour, the Health Belief Model, and Social Cognitive Theory were each used in evaluating a decision aid for postpartum women in Kenya [11] whilst the TFA was not. We found the TFA to be highly applicable to developing middle-range theories for mechanisms of acceptability of FP. We found one adjustment was needed to the wording of one of the TFA constructs, which was the addition of ‘…and believe what they are told about how it works’ in reference to the Intervention Coherence construct (Fig. 3). We considered ‘understanding’ the intervention and how it works as weak in terms of triggering a decision that would drive an outcome [18] similarly to the adage that knowledge does not necessarily trigger behaviour change [45]. To accommodate the mechanisms that we identified empirically, we added a new construct to the TFA. In their preliminary TFA, Sekhon et al. included the construct ‘Ethical Consequences’ defined as ‘associated side effects with the intervention’. During further development, this construct was removed. We added the construct of ‘unintended consequences’ to include both the health-related side effects of MCM use and the negative consequences of being exposed to FP-related stigma.

### Context and transferability of interventions

Complex multi-component interventions necessarily take different forms in different contexts as they interact with people, institutions, and other systems [46]. Decision-makers are tasked with understanding whether interventions that work elsewhere will work in their setting. Here, we recommend that for interventions to be accepted, the context and not the intervention should be the first consideration, with the intervention then designed or selected based on CATs. That is, rather than asking ‘will this intervention work in this context?’ the approach should ask: ‘given this context, which intervention do we need to apply?’

Examples of interventions achieving expected outcomes in one geographic site yet failing to do the same when implemented elsewhere are prevalent in both FP and reproductive health more widely [47, 48]. This non-transferability of effectiveness is most often linked to differences in contexts. That is, the mechanisms that drive outcomes, such as acceptability, are not triggered by the intervention in the prevailing context. We suggest that there is significant potential to reduce the prevalence of unsuccessful interventions and resulting waste of resources through firstly defining CATs and subsequently using them to select appropriate interventions.

### Generating and testing CATs and defining context

A practical approach to using CATs to select and implement appropriate interventions in a particular geographic setting would involve two complementary approaches, which are (1) generating and testing further CATs and (2) defining the context in a project/programme site and selecting an appropriate intervention based on these CATs. Our seven CATs provide the basis for a database of context-linked mechanisms of FP acceptability based on the expanded TFA constructs. Further research to expand the current CAT database as a validated resource for decision-makers would involve testing the CATs in other geographic settings, reviewing the literature for evidence to support or refute each of the CATs, and expanding the database to add new CATs as they are developed from empirical studies. The database would serve as a resource to projects/programmes obviating the necessity to develop new CATs in each site. A simple assessment of context could enable the determination of dominant contextual factors influencing the acceptability and uptake of FP. Such an assessment could take the form of individual interviews or a participatory workshop with key informants that ask what influences the acceptability of FP in the defined sites amongst providers and their clients. This simple empirical investigation of context coupled with reference to the CAT database would then inform the selection of interventions.

Finally, our study focussed on CATs specifically for acceptability and uptake of FP; however, similar CATs could be developed for practical application with other public health interventions using the approach outlined in this manuscript.

Our study made a number of novel contributions, each demonstrating the value of realist approaches in implementation research. We showed that despite the complexity of context, there were a limited number of major contextual factors that influenced mechanisms of FP acceptability in key actors. We highlighted the usefulness of abstracting context-linked empirical mechanisms to constructs of the TFA in predicting what intervention should be implemented where, and critiqued and suggested adaptation of the TFA. We defined context-acceptability cascades which are key to understanding the need to adapt implementation through the life of a project. In terms of the field of FP specifically, whilst the mechanisms of FP acceptability included recognised strategies, they explained why these strategies worked and where they would and would not work. The need for evidence on other more novel strategies such as triggering ethicality in religious leaders and the question of covert MCM use were also identified.

### Limitations

Realist approaches assume that actors will experience an intervention differently depending on their role; thus, their different experiences must be considered and captured. We interviewed respondents with a range of roles in, and relationships to, the interventions in the five countries; however, we were limited in the number of respondents within each role. This introduces a potential limitation in fully capturing some of the variability in how those in the same role perceived the intervention to work. Some differences in contexts and mechanisms across the five countries were not presented here as they centred less on the acceptability of FP in communities and more on the accessibility of services. However, our aim was to collapse findings to look for commonalities rather than highlight differences. We were unable during this study to fully explore the relationship between actors, processes, and interventions. That is, the extent to which involvement in the process of an intervention versus the intervention *per se* triggers a mechanism of acceptability was not central to this investigation. This will be an aim of our future research.

## Conclusion

This study demonstrated the value of aligning realist approaches within implementation research. It is possible to generate CATs by identifying (1) the prevailing contexts that drive the acceptability of FP and MCM use and (2) the interventions that trigger mechanisms of acceptability amongst specific actors within these contexts, and subsequently aligning these mechanisms to constructs of the TFA. CATs are transferable theories that answer the question: given the context, what construct of acceptability does an intervention need to trigger, or more simply, what intervention do we need to apply here to achieve our outcomes?

## Supplementary Information

**Additional file 1.** Standards for Reporting Qualitative Research (SRQR) checklist

## Data Availability

The datasets used and/or analysed during the current study are available from the corresponding author on reasonable request.

## Abbreviations

CATs: Context-acceptability theories
CAMO: Context-actor-mechanism-outcome
CIAMO: Context-intervention-actor-mechanism-outcome
CHWs: Community health workers
CMO: Context-mechanism-outcome
DHS: Demographic and Health Survey
EPI: Expanded Programme on Immunisation
FP: Family planning
FGD: Focus group discussion
HCW: Healthcare worker
IEC: Information, education, and communication
IUD: Intrauterine device
MCMs: Modern contraceptive methods
NGO: Non-governmental organisation
SSI: Semi-structured interview
TFA: Theoretical Framework on Acceptability

## Footnotes

1 Sexually active women 15–49 years of age not using a contraceptive method who report not wanting any more children or wanting to delay their next child.
