# Supplementary material for: Context-acceptability theories: example of family planning interventions in five African countries
Source: Implement Sci. 2021 Jan 12;16:12. doi: 10.1186/s13012-020-01074-z (PMC7805098; doi:10.1186/s13012-020-01074-z)
Supplement: Supplementary file 1 — Additional file 1. Standards for Reporting Qualitative Research (SRQR) checklist [file 13012_2020_1074_MOESM1_ESM.docx]

**Standards for Reporting Qualitative Research (SRQR) checklist**

| **Title and abstract** |  |
| --- | --- |
| 1.Title | Includes a concise description of the nature and topic of the study. P.1 |
| 2. Abstract | Abstract includes background, purpose (aim), methods, results and conclusions p. 2-3 |
| **Introduction** |  |
| 3. Problem formulation | Significance of the problem studied p.4-5; relevant theory (analytical framework) p.8-9, figure 1; empirical work p.4 |
| 4. Purpose or research question | Purpose (aim) p.6 |
| **Methods** |  |
| 5. Qualitative approach and research paradigm | Qualitative approach (realist narrative research – semi-structured interviews, focus group discussions) p.7. Guiding theory (realist) p.5. Analytical framework p.8-9. |
| 6. Researcher characteristics and reflexivity | Researcher characteristics and institutional background p.8 & p.1. |
| 7. Context | Setting and salient contextual features p.6 |
| 8. Sampling strategy | Selection of stakeholders for in-depth interviews and focus group discussions p. 8 |
| 9. Ethical issues pertaining to human subjects | Consent from six ethics committees and informed consent p. 11 |
| 10. Data collection methods | Data collection procedures p. 8 |
| 11. Data collection instruments and technologies | Theme guide details and iterative process p. |
| 12. Units of study | Type of participants p.8; number of participants p.11 + Table 1 |
| 13. Data processing | Data management prior to analysis p. 8 |
| 14. Data analysis | Primary coding framework + initial thematic analysis p.8-9; construction of context-mechanism-outcome configurations p.10; context-acceptability synthesis p.10. |
| 15. Techniques to enhance trustworthiness | Analysis and interpretation conducted by multiple authors to enhance objectivity p. 10 |
| **Results/Findings** |  |
| 16. Synthesis and interpretation | Main findings p.11-16; development of context-acceptability theories p15-16 & Table 4 and a context-acceptability cascade model p.16 & Figure 3. |
| 17. Links to empirical data | Stakeholder quotes Table 2 |
| **Discussion** |  |
| 18. Integration with prior work, implications, transferability, and contributions to the field | Using the Theoretical Framework on Acceptability to allow for abstraction and comment on transferability within contexts and across geographic sites; Highlighting insights on novel family planning strategies ; generating and testing context-acceptability p.17-21. |
| 19. Limitations | Limited in the number of respondents within each stakeholder role. Relationship between actors, processes and interventions requires more exploration. P.21-22. |
| **Other** |  |
| 20. Conflicts of interest | None to declare p.29 |
| 21. Funding | Pfizer Foundation p. 29 |
